# Characterization of the interactions between *Escherichia coli* receptors, LPS and OmpC, and bacteriophage T4 long tail fibers

**DOI:** 10.1002/mbo3.384

**Published:** 2016-06-06

**Authors:** Ayaka Washizaki, Tetsuro Yonesaki, Yuichi Otsuka

**Affiliations:** ^1^ Department of Biological SciencesGraduate School of ScienceOsaka University1‐1 Machikaneyama‐choToyonakaOsaka560‐0043Japan; ^2^ Department of MicrobiologySchool of MedicineDokkyo Medical University880 Kitakobayashi, Mibu‐machiShimotsuga‐gunTochigi321‐0293Japan

**Keywords:** Adsorption, bacteriophage T4, *Escherichia coli*, LPS, ompc, tail fibers

## Abstract

Bacteriophages have strict host specificity and the step of adsorption is one of key factors for determining host specificity. Here, we systematically examined the interaction between the *Escherichia coli* receptors lipopolysaccharide (LPS) and outer membrane protein C (OmpC), and the long tail fibers of bacteriophage T4. Using a variety of LPS mutants, we demonstrated that T4 has no specificity for the sugar sequence of the outer core (one of three LPS regions) in the presence of OmpC but, in the absence of OmpC, can adsorb to a specific LPS which has only one or two glucose residues without a branch. These results strengthen the idea that T4 adsorbs to *E. coli* via two distinct modes, OmpC‐dependent and OmpC‐independent, suggested by previous reports (Prehm et al. 1976; Yu and Mizushima 1982). Isolation and characterization of the T4 mutants Nik (No infection to K‐12 strain), Nib (No infection to B strain), and Arl (altered recognition of LPS) identified amino acids of the long tail fiber that play important roles in the interaction with OmpC or LPS, suggesting that the top surface of the distal tip head domain of T4 long tail fibers interacts with LPS and its lateral surface interacts with OmpC.

## Introduction

Bacteriophages (Phages) are viruses that infect and kill bacteria, but the host range of phages is generally narrow. The limited host range is determined by a variety of bacterial‐resistant mechanisms against phages, such as inhibition of phage adsorption, restriction‐modification system, abortive infection (Abi) system, and clustered regularly interspaced short palindromic repeats (CRISPR)‐associated Cas system (Labrie et al. 2010; Samson et al. 2013). The T4 phage, which is one of the best characterized phages, infects only limited strains of *Escherichia* and *Shigella* (Miller 1949; Jesaitis and Goebel 1953). However, T4 can produce progeny in some bacteria other than *Escherichia* and *Shigella*, such as *Salmonella*,* Aerobacter*,* Proteus*,* Serratia,* and *Yersinia*, by injecting T4 genomic DNA into spheroplasts of these bacteria (Wais and Goldberg 1969; Dawes 1975). Because T4 cannot naturally infect these bacteria, the inability of T4 phage in growth on these bacteria is mainly limited by the step of adsorption. This step is triggered by a process in which phage tail fibers recognize specific receptors on the host cell surface (Goldberg et al. 1994). When T4 adsorbs to *E. coli* cells, two different tail fibers, long and short, are involved. First, at least three of six long tail fibers bind to *E. coli* receptors reversibly. Next, the short tail fibers bind irreversibly to receptors and then the conformation of the baseplate changes from hexagonal to star‐shaped for injection of phage DNA (Simon and Anderson 1967; Wilson et al. 1970; Crawford and Goldberg 1980; Hu et al. 2015). The first process plays an important role in the determination of T4 host range, because Krisch's group isolated T4 mutants changing host specificity and identified that those mutations are located in gene *37*, which encodes the distal tips (DT) of T4 long tail fibers (Tétart et al. 1996). Moreover, crystal structure of the DT region suggested that the DT head domain binds to receptors (Bartual et al. 2010).

Lipopolysaccharide (LPS) and outer membrane protein C (OmpC) are defined as receptors when T4 adsorbs to *E. coli* K‐12 strain (Yu and Mizushima 1982; Montag et al. 1990). LPS is a component of the outer leaflet of the outer membrane in gram‐negative bacteria and is composed of three regions, lipid A, inner core, and outer core, and OmpC is a transmembrane protein in the outer membrane. Mizushima and his colleague examined the requirement of LPS structures for T4 adsorption to K‐12 strain, demonstrating that the removal of sugars in outer core of LPS is still able to support the adsorption of T4 in the presence of OmpC and also that T4 can adsorb to a LPS mutant that has a terminal glucose in outer core in the absence of OmpC (Yu and Mizushima 1982). Another susceptible host is *E. coli* B strain which has a deletion in the genomic sequence for OmpC and T4 uses only an LPS having terminal two glucoses in outer core as a receptor in this strain (Wilson et al. 1970; Prehm et al. 1976; Montag et al. 1990). Thus, T4 exhibits two different modes of adsorption, one of which depends on OmpC and the other is independent of OmpC. These observations suggest that DT can bind to a different type of molecules, sugar chain and protein, or T4 might adsorb to K‐12 and B strains by a common mechanism to bind to LPS, but the difference in LPS structure between these two strains might result in different requirements for OmpC as a cofactor. However, further experiments to verify these possibilities have been hardly performed and this fundamental question remains open. Therefore, how DT recognizes receptors in the presence and absence of OmpC is still unknown.

In this study, we first systematically and thoroughly examined the role of LPS in T4 adsorption dependent on or independent of OmpC using various LPS mutants. Similarly to previous results (Prehm et al. 1976; Yu and Mizushima 1982), the result strongly suggests that T4 long tail fibers bind to OmpC in adsorption dependent on OmpC and to a terminal glucose of outer core in adsorption independent of OmpC. Second, we isolated the T4 mutants Nik (No infection to K‐12 strain), Nib (No infection to B strain), and Arl (Altered recognition of LPS) to identify amino acids in DT required for the recognition of LPS or OmpC. Characterization of these mutants shed a light on understanding the recognition of different types of molecules by T4 long tail fiber.

## Experimental Procedures

### *E. coli* strains and phages

*Escherichia coli* strains used in this study are listed in Table 1. Wild‐type phage is T4D (Doermann and Hill 1953).

**Table 1:** *E. coli* strains used in this study

Strains	Genotype	Source/Reference
BW25113	*rrnB3* ∆*lacZ4787 hsdR514* ∆(*araBAD*)*567* ∆(*rhaBAD*)*568 rph‐1*	NBRP‐*E. coli* at NIG
JW3601	BW25113 ∆*waaR*::*kan*	NBRP‐*E. coli* at NIG
JW3602	BW25113 ∆*waaO*::*kan*	NBRP‐*E. coli* at NIG
JW3606	BW25113 ∆*waaG*::*kan*	NBRP‐*E. coli* at NIG
TY0703	BW25113 ∆*waaO‐waaB*::*cm*	This study
TY0707	BW25113 ∆*waaF*::*cm*	This study
TY0708	BW25113 ∆*waaC*::*cm*	This study
JW2203	BW25113 ∆*ompC*::*kan*	NBRP‐*E. coli* at NIG
TY0721	BW25113 ∆*waaR* ∆*ompC*::*kan*	This study
TY0722	BW25113 ∆*waaO* ∆*ompC*::*kan*	This study
TY0723	BW25113 ∆*waaO‐waaB::cm* ∆*ompC*::*kan*	This study
TY0726	BW25113 ∆*waaG* ∆*ompC*::*kan*	This study
TY0727	BW25113 ∆*waaF*::*cm* ∆*ompC*::*kan*	This study
TY0728	BW25113 ∆*waaC*::*cm* ∆*ompC*::*kan*	This study
BB	B strain wild‐type *sup* ^*0*^	Kai et al. 1999;
B40su1	B strain *supD*	Kai et al. 1999;
O157:H7	Wild‐type (ATCC43888)	Morita et al. 2002;
TY0731	O157:H7 ∆*waaI*::*cm*	This study
TY0732	O157:H7 ∆*waaJ*::*cm*	This study
TY0733	O157:H7 ∆*per*::*cm*	This study
TY0750	O157:H7 ∆*ompC*::*kan*	This study
TY0751	O157:H7 ∆*waaI*::*cm* ∆*ompC*::*kan*	This study
TY0752	O157:H7 ∆*waaJ*::*cm* ∆*ompC*::*kan*	This study

### Construction of a plasmid

To clone *E. coli* K‐12 *ompC*, a DNA fragment containing *ompC* was amplified by polymerase chain reaction (PCR) using *E. coli* W3110 DNA as the template and the primers 5′‐CCGGTACCTAAAAAAGCAAATAAAGGCA and 5′‐CCAAGCTTTGTACGCTGAAAACAATG, digested with *Kpn*I and *Hin*dIII and ligated into the corresponding sites of pBAD33 (Guzman et al. 1995) to construct pBAD33‐*ompC*_K‐12_.

### Bacteriophage adsorption assay

*E. coli* cells were grown in Luria–Bertani (LB) medium at 37°C until the density reached 3 × 10^8^ cells mL^−1^ and T4 was added at a multiplicity of infection (m.o.i) of 0.01. At appropriate times after phage addition, an aliquot was withdrawn and diluted with BS buffer (8% NaCl and 10 mmol/L potassium phosphate buffer, pH 7.4) saturated with chloroform. The number of unadsorbed phage particles was determined by plating with BW25113 for T4 wild‐type and Nib or BB for Nik as an indicator cell. The fraction of unadsorbed phage particles was calculated with the number of input phage particles set to 100%. Adsorption assays were performed three times independently and data points represent the mean ± SD of triplicate measurements.

### Purification of lipopolysaccharides (LPS) from *E. coli*

Dried *E. coli* cells were prepared according to the method of Inagaki et al. (1995) with slight modification. Briefly, 7 L of overnight cultures of *E. coli* cells in LB medium was centrifuged at 4300*g* for 15 min and cell pellets were washed successively with 10–15 mL of ethanol, acetone, and 30–45 mL of diethyl ether. After washing, cells were dried under a reduced pressure over SiO_2_·nH_2_O for 2 or 3 days. LPS was extracted from the dried cells by the phenol‐chloroform‐petroleum ether method (Galanos et al. 1969), and dissolved with 50 mmol/L Tris‐HCl (pH 7.0). To check the integrity and purity of extracted LPS, LPS was separated by Tricine‐sodium dodecyl sulfate polyacrylamide gel electrophoresis (SDS‐PAGE) through 20% gels as described previously (Schägger 2006) and visualized by silver staining.

### Inactivation assay of T4 phage by purified LPS

Different concentrations of LPS were mixed with 3 × 10^7^ particles of phage in 1 mL of M9C medium (M9‐glucose medium supplemented with 0.3% casamino acids, 1 *μ*g mL^−1^ thiamine and 20 *μ*g mL^−1^ tryptophan) containing 25 mmol/L Tris‐HCl (pH 7.0). At 10 min after phage addition, the mixture was diluted with BS buffer and the number of infective phage particles was determined by plating with appropriate cells. The relative numbers of infective phage particles in different LPSs was calculated with the number of infective phage particles in the absence of LPS set to 100%. Experiments were performed three times independently and data points represent the mean ± SD of triplicate measurements.

### Isolation of Nik‐, Nib‐, and Arl‐mutant phages

To isolate Nik‐mutant phage which have lost the ability to absorb to K‐12 cells, four cycles of subtraction were performed using a stock of T4 phage particles prepared on BB cells as follows: BW25113 cells were incubated in 100 mL of LB medium at 37°C until the cell density reached 1 × 10^8^ mL^−1^, collected by centrifugation at 2300*g* for 5 min, and suspended with 0.8 mL of fresh LB medium. The suspension was incubated with 1.2 mL of Dil2 buffer (0.01 mol/L Tris‐HCl pH 7.5, 1 mmol/L MgCl_2_, 0.5% NaCl, 0.001% gelatin) containing 10^11^ particles of T4 for 5 min at 37°C. To remove adsorbed phage particles, *E. coli* cells were killed by addition of chloroform and centrifuged at 4400*g* for 5 min. The supernatant containing unadsorbed phage particles was used for a second cycle of subtraction. In the second cycle, 10^10^ cells of growing BW25113 cells concentrated in 18 mL of LB medium by centrifugation were mixed with 2 mL of the supernatant after the first cycle of subtraction. After incubation at 37°C for 5 min, unadsorbed phage particles were again recovered by centrifugation, precipitated with polyethylene glycol as described previously (Yamamoto et al. 1970), and suspended with 1 mL of Dil2 buffer. In the third cycle of subtraction, 1 mL of the suspension after the second cycle of subtraction was incubated with 0.5 mL of LB containing 10^10^ cells of growing BW25113 at 37°C for 5 min. After centrifugation, the supernatant containing unadsorbed phage particles was subjected to the fourth cycle of subtraction. Finally, 1.5 mL of the stock solution containing unadsorbed phage particles (5.4 × 10^8^ mL^−1^) was obtained. After plating the stock solution with BB cells as an indicator, phage plaques were recovered and transferred onto plates seeded with BB or BW25113 cells. 0.3% of phage particles in the stock solution could not form a plaque on BW25113 cells. Nib‐mutant phage was isolated by the same method as for Nik‐mutant phage except that BW25113 cells were substituted for BB cells. Finally, 1 mL of the stock solution of Nik (2.6 × 10^6^ mL^−1^) was obtained. 26.2% of phage particles in the stock solution could not form a plaque on BB cells. Arl‐mutant phage was isolated as follows: When 1.7 × 10^7^ pfu of T4 phage were plated with TY0722 (∆*waaR* ∆*ompC*) as an indicator, 147 faint, small plaques were formed. We randomly picked up four plaques, and those phages were plated with TY0722 cells again. Next, a single bigger and clearer plaque from each clone was utilized to prepare a high‐titer stock after propagation on TY0722 in M9C medium and we obtained a high‐titer stock of three clones out of four original isolates. Sequence analysis for the DT regions of Nik, Nib, and Arl mutants were performed using two primers, 5′‐AAGTCCGCATATCCAAAGTTAGCTGTTGC and 5′‐TATATTTTCATATTTAGAAGGGCCGAAGC.

## Results

### T4 adsorption dependent on OmpC

**Figure 1:**
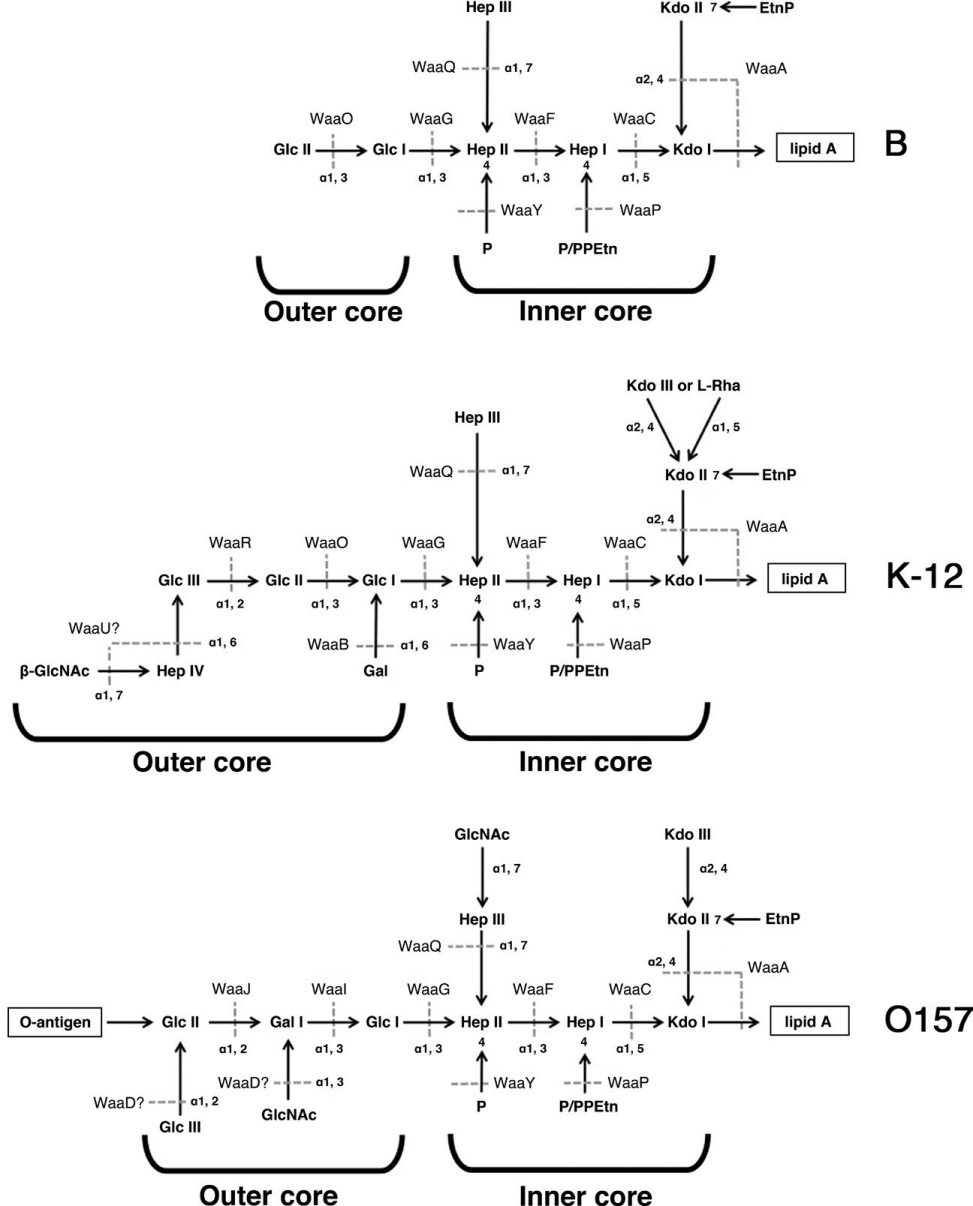
Structures of lipopolysaccharide (LPS) in *E. coli* B, K‐12, and O157 strains. Structures of LPS in B (top panel), K‐12 (middle panel), and O157 (bottom panel) strains are shown. Horizontal and vertical arrows indicate a main chain and sugar branches, respectively. Dotted lines show the reactions catalyzed by sugar transferases. The abbreviations used in the figure are; Glc Glucose; Gal, Galactose; GlcNAc, N‐acetylglucosamin; Kdo, 3‐deoxy‐D‐manno‐oct‐2‐ulosonic acid; Hep, L‐glycero‐D‐manno heptose; Rha, Rhamnose; EtnP, ethanolamine phosphate; PPEtn, 2‐aminoethyl diphosphate; and P, Phosphate.

**Figure 2:**
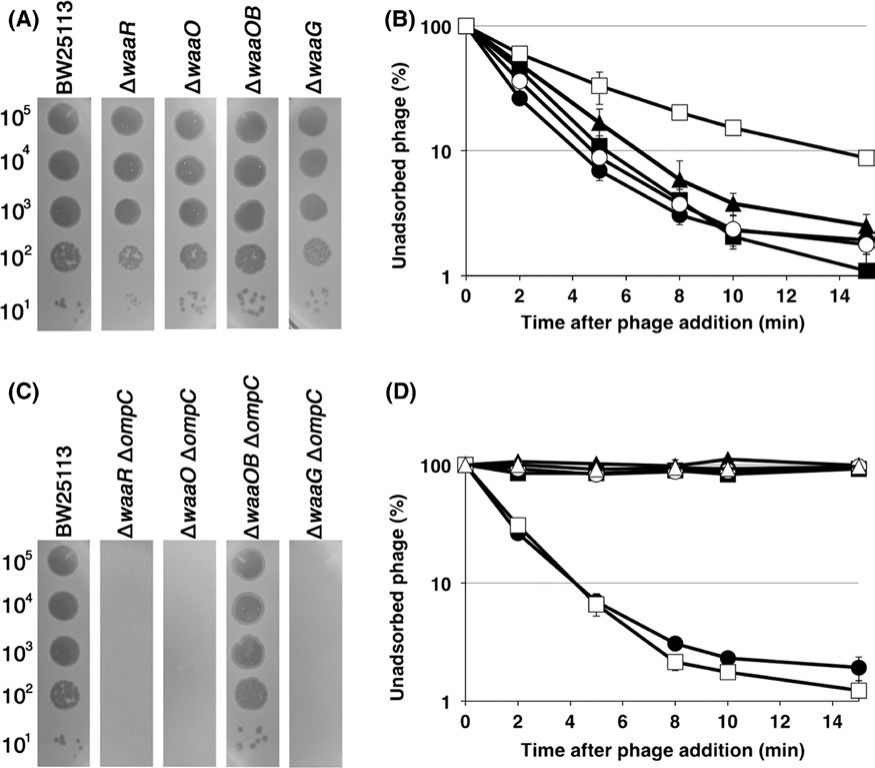
Growth and adsorption of T4 phage on K‐12 outer‐core mutants. (A and C) The solution containing the number of T4 phage particles indicated on the left was spotted on a lawn of the *E. coli* strain indicated at the top and the plates were incubated at 37°C overnight. (B and D) Adsorption analyses were performed as described in Experimental Procedures. Symbols for (B): ●, BW25113; ■, ∆*waaR*; ▲, ∆*waaO*; ○, ∆*waaOB*; □, ∆*waaG*. Symbols for (D): ●, BW25113; ■, ∆*ompC*; ▲, ∆*waaR* ∆*ompC*; ○, ∆*waaO* ∆*ompC*; □, ∆*waaOB* ∆*ompC*; △, ∆*waaG* ∆*ompC*.

T4 phage uses LPS and OmpC to adsorb to the *E. coli* K‐12 strain (Henning and Jann 1979). LPS is composed of three regions, lipid A, inner core, and outer core. Lipid A is mostly buried in a lipid bilayer of the outer membrane. The inner core is an oligosaccharide attached to the extracellular side of lipid A, and the outer core is also an oligosaccharide extended from the inner core. While the structures of Lipid A are highly conserved among *E. coli* strains, those of the inner core and the outer core are diverse and are classified into five types (Schnaitman and Klena 1993; Heinrichs et al. 1998b; Amor et al. 2000). The outer core of K‐12 LPS is a K‐12 type whose main chain is composed of Glc I, Glc II, and Glc III (Fig. 1). Branches of this strain are Gal attached to Glc I and Hep IV‐GlcNAc to Glc III. We first attempted to identify which structure of LPS is required for T4 adsorption dependent on OmpC. For this purpose, we employed a variety of sugar transferase‐deficient mutants (Fig. 1). WaaO is the Glc II transferase and WaaB is the Gal transferase. Therefore, these deletion mutants, JW3602 (∆*waaO*) and TY0703 (∆*waaOB*), synthesize Glc I branched with Gal and only Glc I in the outer core, respectively. WaaG is the Glc I transferase and JW3606 (∆*waaG*) completely eliminates the outer core (Heinrichs et al. 1998b). ∆*waaR* lacks Glc III transferase and its outer core is expected to have two forms, one with Glc II at the outer terminus and the other with GlcNAc‐Hep IV branched to Glc II (Pradel et al. 1992). The LPS synthesized in each mutant was extracted and analyzed by Tricine‐SDS‐PAGE and silver staining (Fig. S1). In this analysis, the mobility of LPS depends on the mass of the polysaccharide and the relative migration rate of each LPS was consistent with its mass. LPS isolated from ∆*waaR* revealed two bands and their migration rates were consistent with the above notion. First, we carried out spot‐test analysis to test the growth ability of T4 on these LPS mutants (Fig. 2A). T4 grew normally on all outer‐core mutants at an efficiency of plating similar to that on wild‐type. Next, the rate of T4 adsorption to each mutant was measured by plaque‐forming assays. After T4 was added to *E. coli* cultures, an aliquot at appropriate times was treated with chloroform and the number of phage particles was counted by plating with BW25113 (K‐12 wild‐type) cells as an indicator. Since chloroform kills *E. coli* cells including those infected with T4, only unadsorbed phages can form plaques. Therefore, the reduction in plaque number along with time reflects the kinetics of T4 adsorption. As shown in Figure 2B, T4 adsorbed efficiently to all outer‐core mutants, although the efficiency was slightly lower in ∆*waaG* compared to the other mutants. This observation suggested that T4 had no specificity for the sugar sequence of the outer core in the presence of OmpC. To further address this possibility, we adopted *E. coli* strain O157. The outer core of the O157 LPS is quite different from those of K‐12 and B strains and belongs to the R3 type in which the main chain of the outer core is Glc I, Gal I, and Glc II with GlcNAc branch attached to Gal I and Glc III branch to Glc II (Fig. 1). In addition, O157 LPS has repetitive units of oligosaccharides (O‐antigen) on the extracellular side of the outer core (Currie and Poxton 1999; Shimizu et al. 1999). O157 has OmpC and its amino acid sequence shares 94% homology with that of K‐12 OmpC. T4, however, cannot use O157 OmpC as a receptor for adsorption (unpublished data). Therefore, we introduced a plasmid expressing K‐12 OmpC into two O157 strains carrying a deletion of *ompC*, TY0750 (O157 ∆*ompC*) and TY0752 (O157 ∆*waaJ* ∆*ompC*). ∆*waaJ* eliminated O‐antigen, leaving Glc I and Gal I branched with GlcNAc in the outer core. T4 could adsorb to O157 ∆*waaJ* ∆*ompC* expressing K‐12 OmpC as efficiently as to K‐12 wild‐type, but hardly at all to O157 ∆*ompC* with K‐12 OmpC (Fig. 4A). This result strengthened the idea that T4 has no specificity for the sugar sequence of the outer core in OmpC‐dependent adsorption. Also, O‐antigen is strongly suggested to function as an inhibitor of T4 adsorption.

**Figure 3:**
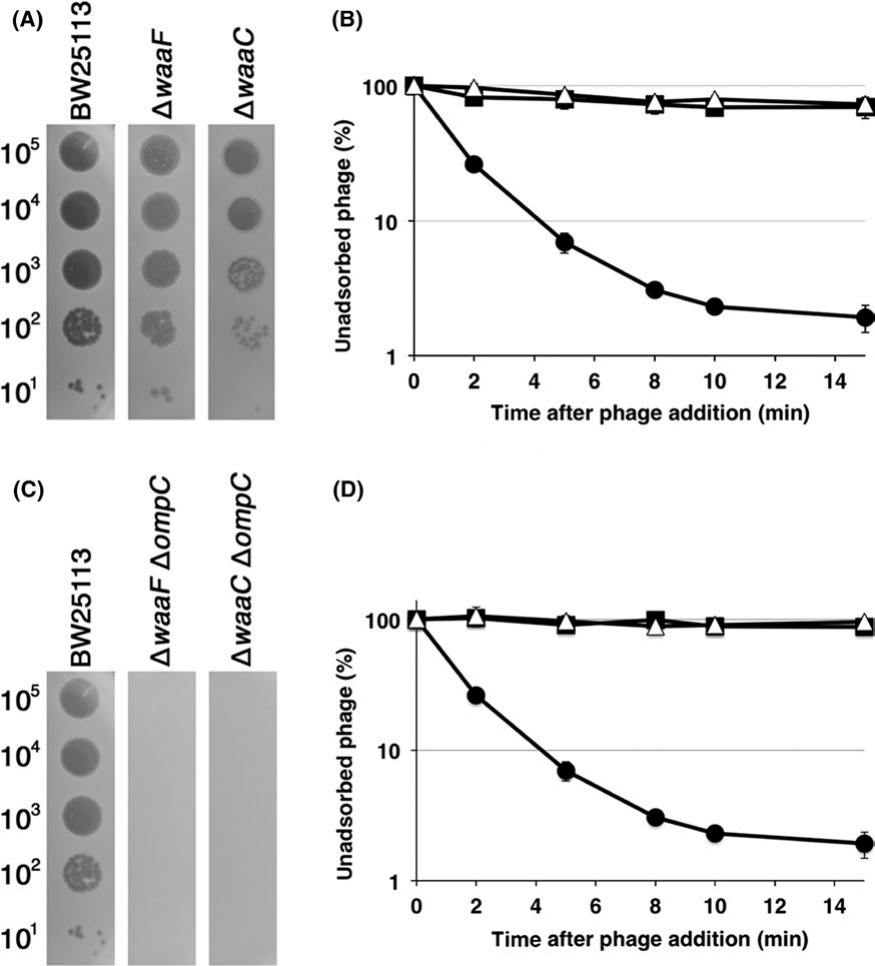
Growth and adsorption of T4 phage on K‐12 inner‐core mutants. (A and C) The solution containing the number of phage particles indicated on the left was spotted on a lawn of the *E. coli* strain indicated at the top and the plates were incubated at 37°C overnight. (B and D) Adsorption analyses were performed as described in Experimental Procedures. Symbols for (B): ●, BW25113; ■, ∆*waaF*; △, ∆*waaC*. Symbols for (D): ●, BW25113; ■, ∆*waaF* ∆*ompC*; △, ∆*waaC* ∆*ompC*.

Next, we examined the necessity of the inner core for T4 adsorption. WaaF and WaaC are Hep II and Hep I transferases, respectively. Therefore, ∆*waaF* and ∆*waaC* lack two or three heptoses in the inner core (Fig. 1). T4 formed plaques on these mutants at an efficiency of plating similar to that on wild‐type, although they were more faint than those on wild‐type (Fig. 3A). Surprisingly, T4 could not adsorb to these mutants efficiently and only 30% of phages adsorbed within 15 min after phage addition (Fig. 3B). This inefficient adsorption may cause the faintness of plaques on these mutants. This result demonstrates that intact inner core is necessary for efficient adsorption of T4 in the presence of OmpC.

### T4 adsorption independent of OmpC

**Figure 4:**
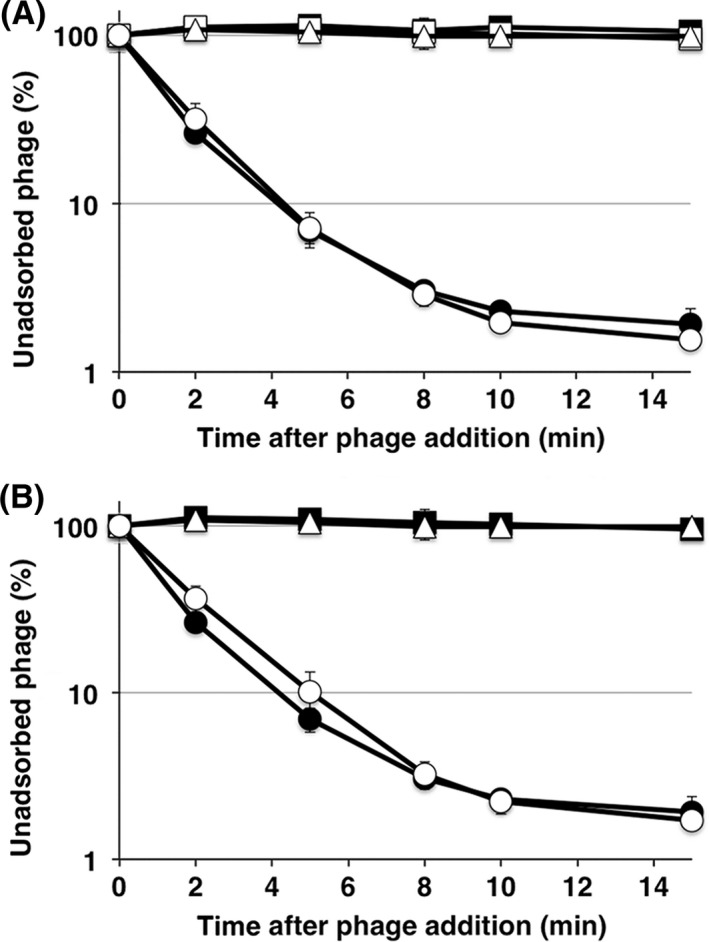
Adsorption of T4 phage on O157 LPS mutants. (A) *E. coli* mutants harboring pBAD33‐*omp* *C*_K__‐12_ were incubated in LB medium until the OD_600_ reached 0.3, and arabinose was added to a final concentration of 0.2% for 30 min. Adsorption analyses were performed as described in Experimental Procedures. Symbols: ●, BW25113; ■, O157; △, O157 ∆*waaJ*; ○, O157 ∆*waaJ* ∆*ompC* harboring pBAD33‐*ompC*_k‐12_; □, O157 ∆*ompC* harboring pBAD33‐*ompC*_k‐12_. (B) Adsorption analyses were performed as described in Experimental Procedures. Symbols: ●, BW25113; ■, O157; △, O157 ∆*waaJ*; ○, O157 ∆*waaI*. LB, Luria–Bertani; LPS, lipopolysaccharide.

T4 efficiently adsorbs to strain B in which OmpC is not expressed, and previous work demonstrated that T4 uses LPS alone for adsorption to this strain (Wilson et al. 1970; Montag et al. 1990). Although the core oligosaccharide of strain B is of the R1 type, the main chain of which is composed of Glc I, Glc II, and Gal I, B strains used for T4 adsorption have only two glucoses, Glc I and Glc II, in the outer core (Fig. 1), because of an IS*1* insertion in *waaT* encoding Gal I transferase (Heinrichs et al. 1998a; Jeong et al. 2009). Also, T4 can adsorb to K‐12 mutant that has only glucose in outer core without OmpC (Yu and Mizushima 1982). Therefore, this specific type of LPS functions as a receptor for T4 adsorption without OmpC. To reevaluate which structure of LPS supports T4 adsorption in the absence of OmpC, *ompC* was deleted from K‐12 wild‐type and LPS mutants. T4 grew on and adsorbed to TY0723 (∆*waaOB* ∆*ompC*) efficiently, but not on other LPS mutants without OmpC (Fig. 2C and D). The LPS of ∆*waaOB* ∆*ompC* has an outer core with only Glc I, similarly to the B strain, and this result is consistent with the previous result (Yu and Mizushima 1982). Interestingly, when only a galactose was branched to Glc I in TY0722 (∆*waaO* ∆*ompC*), efficient adsorption of T4 disappeared. Furthermore, when all of the outer core and parts of the inner core were removed, T4 could neither form plaques nor adsorb (Fig. 3C and D). Also, T4 adsorbed to an O157 LPS mutant (O157 ∆*waaI*, which has only Glc I in the outer core), as efficiently as to the K‐12 strain (Fig. 4B). These results indicate that T4 can bind to a specific LPS with only one (Glc I) or two glucoses (Glc I and Glc II) in the outer core, regardless of the sugar sequences in the inner core.

**Figure 5:**
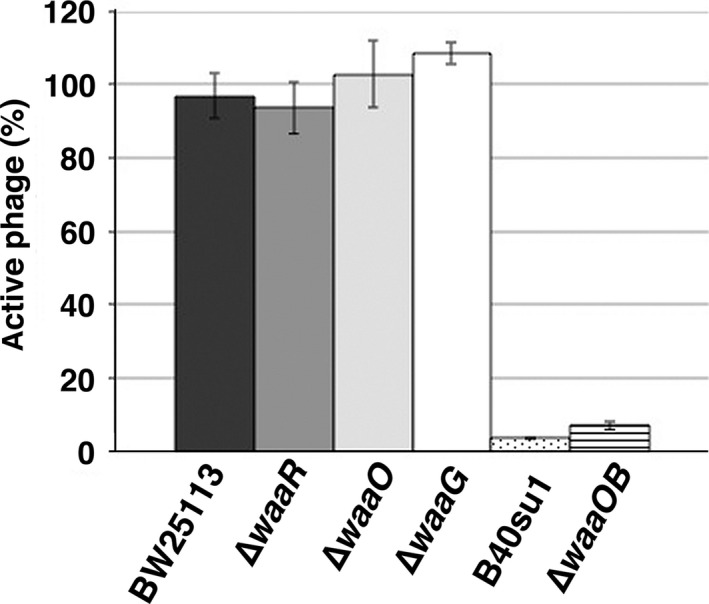
Inactivation assay of T4 phage using purified lipopolysaccharide (LPS). A solution containing 3 × 10^7^ T4 phage particles was mixed with 35 *μ*mol/L of LPS purified from the *E. coli* strains indicated at the bottom and then the inactivation assay was performed as described in Experimental Procedures.

To further examine whether T4 can directly use LPS as a receptor in the absence of OmpC, we performed the inactivation assay using LPS purified from a variety of *E. coli* mutants. When at least three of six long tail fibers of T4 bind to *E. coli* receptors, the conformation of the baseplate changes from hexagonal to star‐shaped and then the short tail fibers bind irreversibly to LPS (Simon and Anderson 1967; Wilson et al. 1970; Crawford and Goldberg 1980; Hu et al. 2015). After this conformational baseplate change, T4 is inactivated or can never adsorb to newly encountered *E. coli* strains. When T4 was mixed with LPS purified from a B strain (B40su1), 95% of input phage particles were inactivated within 10 min (Fig. 5), strongly suggesting that the B strain LPS interacts with T4 tail fibers. On the other hand, the LPS of wild‐type, ∆*waaR*, ∆*waaO,* or ∆*waaG* strain could not inactivate T4 at all. Remarkably, the LPS of ∆*waaOB*, to which T4 could adsorb without OmpC (Fig. 2D), inactivated more than 90% of input particles. These results strongly suggest that T4 can adsorb directly to specific LPS (Glc I or Glc I‐Glc II in outer core).

### Isolation and characterization of T4 Nik and Nib mutants

**Figure 6:**
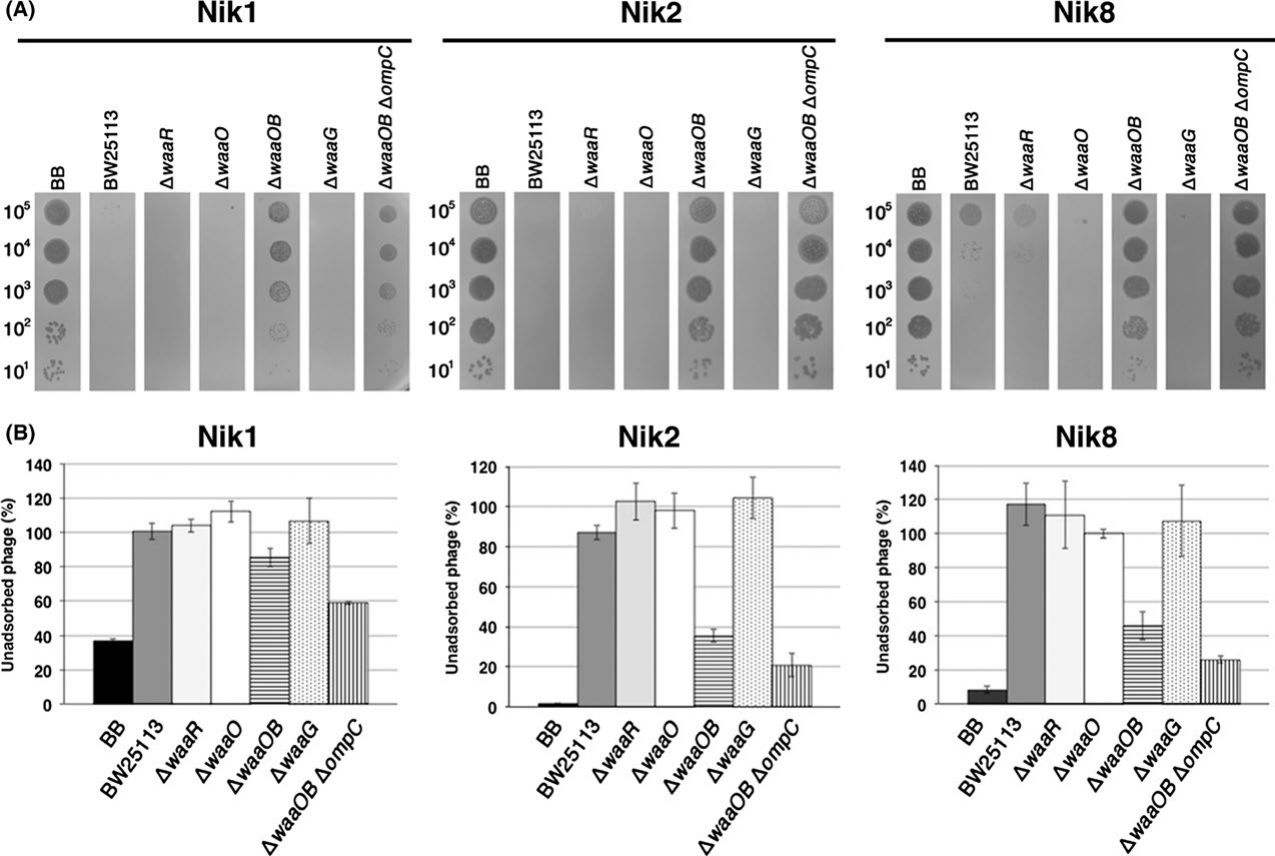
Characterization of T4 Nik mutants. (A) The solution containing the number of phage particles (Nik1, left; Nik2, middle; Nik8, right panel) indicated on the left was spotted on a lawn of the *E. coli* strain indicated at the top and the plates were incubated at 37°C overnight. (B) Adsorption analyses of Nik1 (left), Nik2 (middle), or Nik8 (right) phages with the *E. coli* strain indicated at the bottom were performed. The relative numbers of unadsorbed phage particles at 10 min after phage addition were calculated with the numbers of input phage particles set to 100%.

As demonstrated above, there are two different modes in T4 adsorption, OmpC‐dependent and OmpC‐independent. Previous studies demonstrated that DT binds to receptors on the cell surface (Montag et al. 1990). To identify essential amino acids in the DT region for each mode of adsorption, we attempted to isolate T4 mutants defective in each mode of adsorption. For this purpose, we used K‐12 and B strains because T4 absorbs to strain K‐12 dependent on OmpC and to strain B independent of OmpC. We obtained three Nik (No infection to K‐12 strain) mutants (1, 2, and 8) that grew on B cells but not on K‐12 cells, and 5 Nib (No infection to B strain) mutants that grew on K‐12 cells but not on B cells (see Experimental Procedures). Sequence analysis around the DT region of these mutants identified amino acid substitutions in the DT region. Although we did not examine the sequence of other regions except for DT, marker rescue analysis confirmed that these substitutions in DT caused the changes in host range of T4 adsorption (Table S1). Nik1, 2 and 8 had single‐base substitutions at T2822A, C2864A, and G2827A of gene *37*, replacing amino acids at V941E, A955E, and G943S, respectively (Fig. 9A). None of the Nik mutants could form plaques efficiently on BW25113 (Fig. 6A). While Nik2 and 8 formed normal plaques on BB, K‐12 ∆*waaOB*, and ∆*waaOB* ∆*ompC* cells, Nik1 formed smaller and more faint plaques on BB, ∆*waaOB* and ∆*waaOB* ∆*ompC* cells than plaques formed by Nik 2 and Nik8. Importantly, no Nik mutants could grow on any other LPS mutants, even those expressing OmpC, suggesting the loss of OmpC‐dependency. To confirm this possibility, the OmpC‐requirement for the adsorption of Nik mutants was examined (Fig. 6B). All Nik mutants adsorbed to BB, ∆*waaOB*, and ∆*waaOB* ∆*ompC* cells, although the efficiencies of Nik1 on these strains were lower than those of other Nik mutants. As expected, Nik mutants did not adsorb to any other strains expressing OmpC, indicating that the Nik mutants lose the ability of adsorption dependent on OmpC.

**Figure 7:**
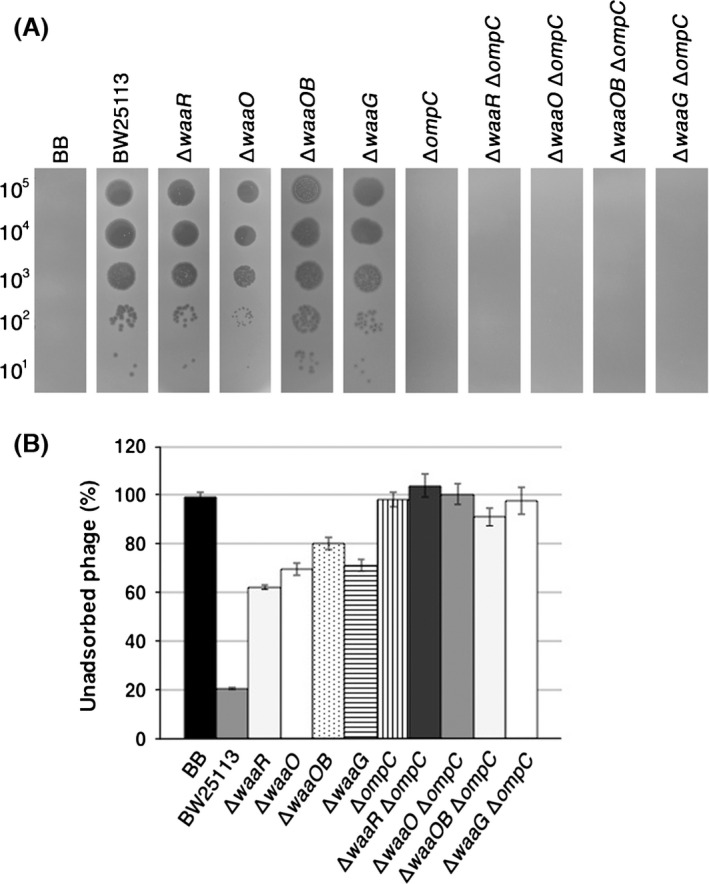
Characterization of the T4 Nib mutant. (A) A solution containing the number of Nib phage particles indicated on the left was spotted on a lawn of the *E. coli* strain indicated at the top and the plates were incubated at 37°C overnight. (B) Adsorption analyses with the *E. coli* strains indicated at the bottom were performed as described in Experimental Procedures.

Although we isolated five Nib mutants, all of them had the same base substitution at C2816T of gene *37*, causing the amino acid substitution T939I. As shown in Figure 7A, Nib formed plaques on K‐12 wild‐type and LPS outer‐core mutants, but not on strain B or any K‐12 ∆*ompC* strain. Adsorption assays showed that Nib adsorbed, depending on OmpC, to all LPS outer‐core mutants, though more than 60% of phage particles remained unadsorbed at 10 min after phage addition (Fig. 7B). On the other hand, Nib hardly adsorbed to the same LPS outer‐core mutants in the absence of OmpC. Notably, Nib could not adsorb to ∆*waaOB* ∆*ompC* in addition to strain B, indicating that Nib lost the ability of OmpC‐independent adsorption and that T939 is responsible for the interaction to terminal glucoses (Glc I and Glc I‐Glc II) in the outer core. These results strongly suggest that DT can bind to both OmpC and LPS, and the different amino acids of DT bind them, respectively.

### Isolation and characterization of T4 Arl mutant

**Figure 8:**
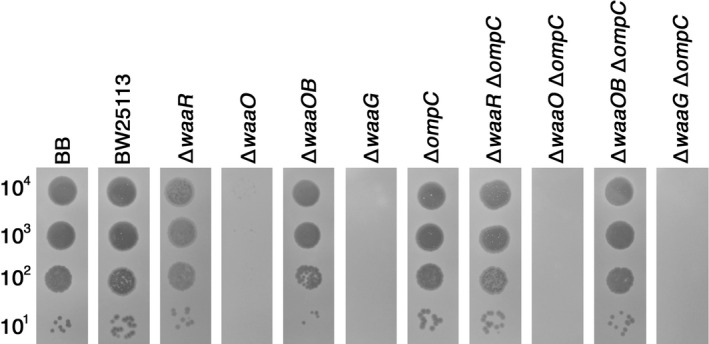
Growth of the T4 Arl mutant. A solution containing the number of Arl phage particles indicated on the left was spotted on a lawn of the *E. coli* strain indicated at the top and the plates were incubated at 37°C overnight.

**Figure 9:**
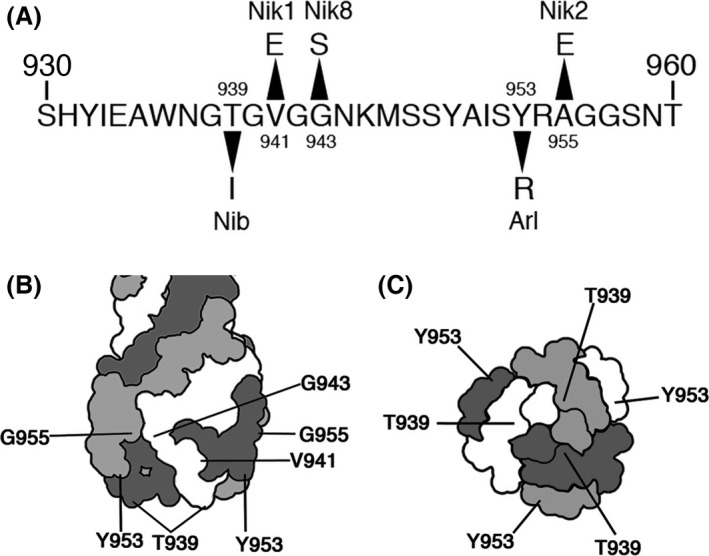
Mutation sites of Niks, Nib, and Arl. (A) Amino acid sequence of the distal tips (DT) region in gp37. Arrowheads indicate substitutions of amino acids in Nik1, 2, 8, Nib, and Arl mutants. Schematic diagrams of the DT region viewed from the lateral surface (B) and the top surface (C) are shown. Lines indicate positions of Niks, Nib, and Arl mutations.

To further identify the DT region involved in binding to LPS, we attempted to isolate Arl (altered recognition of LPS) mutant that can recognize LPS which wild‐type T4 cannot do. For this purpose, we looked for T4 mutants that could grow on ∆*waaR* ∆*ompC*. As a result, three Arl mutants were isolated (see Experimental Procedures). Sequence analyses demonstrated that all three had the same two base substitutions at T2857C and A2858G of gene *37*, causing one amino acid substitution, Y953R (Fig. 9A). The two base changes would probably have arisen by successive mutational events, because the originally isolated mutant formed small, faint plaques while the finally obtained Arl formed normally sized clear plaques on ∆*waaR* ∆*ompC*. Arl could grow on BB, BW25113, ∆*ompC*, ∆*waaR*, ∆*waaOB*, ∆*waaR* ∆*ompC,* and ∆*waaOB* ∆*ompC*, but not on ∆*waaO*, ∆*waaG*, ∆*waaO* ∆*ompC,* and ∆*waaG* ∆*ompC* (Fig. 8). These results strongly suggest that Arl is a gain‐of‐function mutant to recognize the outer cores of K‐12 wild‐type and ∆*waaR* in addition to those of strain B and ∆*waaOB*, and that Y953R is involved in binding to LPS of K‐12 wild‐type and ∆*waaR*.

## Discussion

The requirements of LPS and OmpC for T4 adsorption were established decades ago and two different modes of adsorption, one of which depends on OmpC and the other is independent of OmpC, have been proposed (Prehm et al. 1976; Yu and Mizushima 1982). In this study, we performed a comprehensive analysis of LPS structure in relation to each mode of T4 adsorption using various LPS mutants. All tested outer‐core mutants of strain K‐12 allowed T4 adsorption depending on OmpC (Fig. 2B). T4 was also able to adsorb to O157 ∆*waaJ* ∆*ompC* expressing K‐12 OmpC (Fig. 4A). These results indicate that T4 has no specificity for outer‐core structure in OmpC‐dependent adsorption, although the length of the outer core correlated weakly with the efficiency of adsorption (Fig. 2B). From these results, DT does not seem to bind to LPS and instead, it is likely that DT binds to OmpC directly. OmpC exists as a trimer in the outer membrane. Each OmpC monomer has a pore formed by 16 beta‐barrels and eight loops that connect each *β*‐sheet on the extracellular side (Baslé et al. 2006). The direct binding between OmpC and DT may be supported by our preliminary results; the amino acid substitution at a phenylalanine in the extracellular loop 4 of OmpC caused the inability of T4 adsorption, and this defect was recovered by the mutation of DT (Unpublished data). Also, Trojet et al. (2011) demonstrated that T4‐like phages would use extracellular loops of OmpC for adsorption. Similarly to OmpC, DT is also composed of a trimer of gp37. These facts make us assume that the binding of DT to OmpC requires simultaneous interaction between respective monomers of OmpC and DT. In this connection, LPS seems to play a role in making OmpC functional as a receptor. The previous *in vitro* study showed that OmpC alone could not inactivate T4 and, when both OmpC and LPS were present, a hexagonal lattice was formed on the lipoprotein‐bearing peptidoglycan sacculus, to which T4 could adsorb (Furukawa et al. 1979). Additionally, Buehler et al. (1991) reported that the conductance property of OmpC is affected by LPS and Baslé et al. (2006) reported that the pore geometries in the crystal structure of OmpC correspond to a larger conductance than experimentally measured and its width based on crystal structure is larger than that of DT. Moreover LPS is responsible for assembly of OmpC (Ried et al. 1990). These observations suggest that the width of OmpC is compressed by the surrounding LPS in the outer membrane, rendering OmpC in a form favorable to interaction with DT. The observations that shortening of the inner core, but not the outer core, drastically reduced adsorption (Fig. 3B), and that the difference of inner‐core structure among B, K‐12, and O157 strains hardly affects adsorption (Fig. 2B and 4A) indicate that the intact inner core of LPS is essential for efficient adsorption depending on OmpC and the length of the inner core may be important for compressing OmpC.

Previous studies showed that T4 adsorbed to LPS which has only a glucose in outer core (Yu and Mizushima 1982) and T4 adsorption to the B strain was inhibited by glucose (Dawes 1975). In this study, we reevaluated LPS structure that support T4 adsorption independent of OmpC using not only B and K‐12 strains but also O157 strain. T4 efficiently adsorbed to B and K‐12 ∆*waaOB* in the absence of OmpC (Fig. 2D), and LPS purified from these strains inactivated T4 (Fig. 5). Also, T4 could adsorb to O157 ∆*waaI* in the absence of functional OmpC (Fig. 4B). All these strains commonly have a terminal glucose(s) in outer core. Interestingly, when only a galactose was branched to Glc I in ∆*waaO* ∆*ompC*, T4 could not adsorb (Fig. 2D) and the LPS of this strain was unable to inactivate T4 (Fig. 5). Taken together, a terminal glucose(s) without a branch is an important factor for T4 adsorption independent of OmpC and should be a site of the DT interaction. T4 could not adsorb to ∆*waaR* ∆*ompC* (Fig. 2D). As mentioned in the Result, ∆*waaR* results in two types of LPS. Probably, one of them, the shorter, has a terminal glucose (Glc II) potentially eligible for an interaction with DT. The other, longer type of LPS, presumably has a terminal structure like wild‐type LPS or Glc II attached with GlcNac‐Hep IV. When these two types of LPSs are randomly intermingled on a cell surface at a density of 0.5 LPS molecule nm^−2^ (calculation based on 10^6^ LPS monomers on a cell surface (Smit et al. 1975)), the tip of the shorter LPS would not be able to interact with DT because it is concealed by the longer LPS.

Amino acid substitutions in DT changed the host range (Tétart et al. 1996). However, whether these substitutions are involved in the binding to LPS or OmpC is not determined. Also, Bartual et al. (2010) proposed two docking models of DT and OmpC based on the crystal structure of DT: DT binds to OmpC vertically using the head domain or transversally using the head domain and the needle domain. Isolation and characterization of Nik and Nib mutants indicate that subregions of DT required for binding to OmpC or LPS do not completely overlap. Nik1, 2 and 8 have V941E, A955E, and G943S substitutions, respectively, and all these residues are located on the lateral surface of the DT head domain (Fig. 9B and C). In these mutants, the adsorption dependent on OmpC is inactivated (Fig. 6B). These results suggest that DT binds to the center of an OmpC trimer vertically and that the lateral surface of the DT head domain interacts at multiple sites with the extracellular loops of OmpC. Nib lost its ability to adsorb to the B strain having two glucoses in the outer core and to ∆*waaOB* ∆*ompC* having a single glucose (Fig. 7B). We only obtained one Nib mutant with one amino acid substitution of T939I (Fig. 9A), implicating a narrow interaction site with a terminal glucose in the outer core of LPS. T939 is located at the top of the DT head domain and is flanked with glycines, making the side chain of threonine stand out. In this connection, Krisch and his colleagues reported that a substitution of G938V rendered T4 unable to grow on B strain (Tétart et al. 1996). Valine has a side chain with a branch and is more bulky than glycine. Therefore, it would be reasonable to assume that a valine next to T939 hinders an interaction of T939 to LPS. Therefore, the top surface of the DT head domain containing T939 plays a key role in the recognition of a terminal glucose in the outer core. In this connection, Bartual et al. (2010) lined up some amino acids as candidates to bind to a glucose of LPS based on the crystal structure, and one of these candidates, Y949, is mapped at the top of the DT head domain, although other candidates are not mapped at the top. Y953 in Arl, which became able to adsorb to the LPS of K‐12 wild‐type, but not T939 in Nib, is included in these candidates. Apparently, further study of these candidates in binding to a glucose or other sugars will give more insight into the interaction between DT and LPS.

Arl, which has an amino acid substitution at 953 from tyrosine to arginine, could adsorb to K‐12 ∆*waaOB* in addition to the B strain, like wild‐type T4 did, having a terminal glucose in the outer core (Fig. 8). Surprisingly, the Arl mutant became able to adsorb to strains having LPSs of K‐12 wild‐type and ∆*waaR*. As mentioned above, T4 would not be able to access a terminal glucose of the shorter LPS which ∆*waaR* synthesizes. Thus, it is suggested that Arl recognizes the longer LPS of ∆*waaR* cells, which has GlcNAc and/or Hep at the terminus of outer core like K‐12 wild‐type LPS. The Arl mutant conserves threonine at 939 which is important for recognizing a terminal glucose in the outer core, and it is located at the top of the DT head domain. On the other hand, Y953 is located at the border between the lateral and top surfaces of the head domain (Fig. 9B and C). The side chain of Y953 protrudes toward the top surface (Bartual et al. 2010) and the side chain of arginine replacing tyrosine is also expected to protrude towards the top surface of the head domain. In addition, the side chain of arginine is longer than that of tyrosine. Therefore, this change may enable T4 long tail fibers to interact with the LPSs of K‐12 wild‐type and ∆*waaR* at top surface of the DT, and considering the mutational sites of Nib and Arl mutants, the top surface of the DT would play an important role in binding to LPS.

Antibiotics are now losing potency because of increasing antibiotic resistance and the lack of new types of antibiotics. This situation spotlights a resurgence of phage therapy. However, in contrast to antibiotics that are effective against a wide range of bacterial species, phages infect only a limited range. Because of this strict host specificity, when a new pathogenic bacterium appears, a naturally isolated phage that can infect it is necessary and its safety should be established before use for therapy. This approach takes much time, labor and cost, and isolation of useful phage is not guaranteed. This problem might be solved if we could control the host specificity of a known phage to infect a given strain. Isolation of Arl mutants implies that it is not difficult to change host range by manipulating the structure of the DT head domain, especially the top surface, to recognize various types of LPSs. Since current technology can replace any amino acid, even all, with designated one(s), T4 might be largely expanded to become a tool for phage therapy.

## Conflict of Interest

The authors have no conflict of interest to declare

## Supporting information


**Table S1**. Changes in T4 host range by mutations located in genes *37* and *38* in Nik and Nib mutants
**Figure S1**. Analysis of LPS purified from *E. coli* strains.
