# Supplementary material for: Characterization of the interactions between Escherichia coli receptors, LPS and OmpC, and bacteriophage T4 long tail fibers
Source: Microbiologyopen. 2016 Jun 6;5(6):1003–15. doi: 10.1002/mbo3.384 (PMC5221442; doi:10.1002/mbo3.384)
Supplement: Supplementary file 1 — Table S1. Changes in T4 host range by mutations located in genes 37 and 38 in Nik and Nib mutants Figure S1. Analysis of LPS purified from E. coli strains. [file MBO3-5-1003-s001.pdf]

Supporting Information

Characterization of the interactions between *Escherichia coli* receptors, LPS and OmpC, and bacteriophage T4 long tail fibers

Ayaka Washizaki, Tetsuro Yonesaki and Yuichi Otsuka

List of Contents:

Table S1: Changes of T4 host range by mutations located in genes *37* and *38* in Nik and Nib mutants.

Figure S1: Analysis of LPS purified from *E. coli* strains.

Experimental Procedures: Construction of *E. coli* mutants.

Experimental Procedures: Plasmid construction and marker-rescue test.

References:

Table S1. Changes of T4 host range by mutations located in genes *37* and *38* in Nik and Nib mutants.

Plasmid used for Marker-rescue test	Emergence rate （%）
pLTF	< 0.02
pLTF-Nik1	0.42
pLTF-Nik2	0.90
pLTF-Nik8	0.57
pLTF-Nib	4.7

Marker-rescue test was performed as described in Experimental procedures. The emergence rate (%) shows the ratio of the number of phage plaques showing same host range as Nik or Nib to the total number of phage plaques. Each value indicates the mean of three independent experiments.

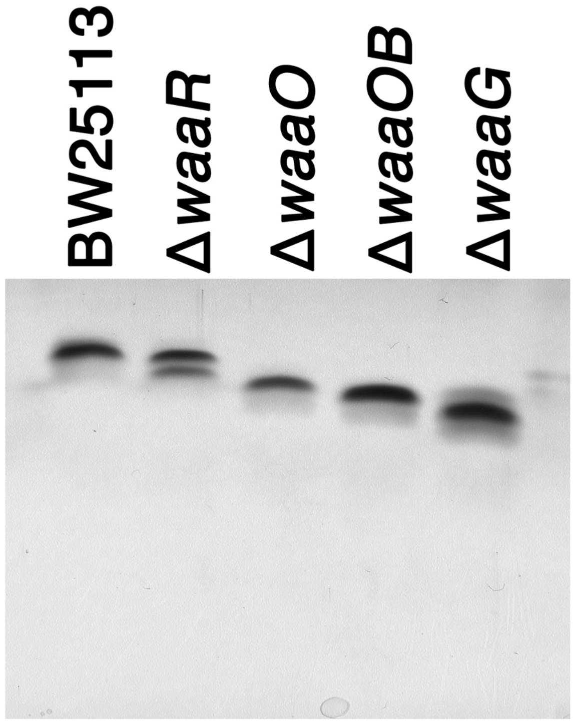

Figure S1. Analysis of LPS purified from *E. coli* strains.

120 μg of LPS purified from the *E. coli* strain indicated at the top was analyzed by 20% Tricine-SDS gel and silver staining.

Experimental Procedures:

Construction of E. coli mutants

Deletion mutants of *ompC* were constructed as follows. First, the kanamycin-resistance cassette was removed from JW3601, JW3602 and JW3606 by yeast Flp recombinase expressed from pCP20 (Cherepanov and Wackernagel, 1995). Next, Δ*ompC*::*kan* was transferred into the above cells and the others by T4 GT7 phage transduction of the kanamycin-resistance cassette from JW2203 (Wilson *et al.*, 1979). TY0703, TY0707 and TY0708 were constructed as described (Datsenko and Wanner, 2000). Briefly, a fragment containing a chloramphenicol-resistance cassette flanked with the sequences upstream and downstream of the gene which should be deleted was amplified by PCR with pKD3 as a template. Primers used for PCR were: for TY0703,
5′-GTCAACGATTGTTTCTGATTTTATAGACAAATAAAACCGTTAAAACAGTGGTG
TAGGCTGGAGCTGCTTC and
5′-TTATATCATTACTTTATAGTTTCCCAGTTTTAATGCTTTATCTTTTCAATATGGG
AATTAGCCATGGTCC; for TY0707,
5′-TAACGGAATACATGGCCTGGCTGAATCGCGACGCATAAGAGCTCTGCATGGT
GTAGGCTGGAGCTGCTTC and
5′-GATGTTTTAACGATCAAAACCCGCATCCGTCAGGCTTCCTCTTGTAACAAATG
GGAATTAGCCATGGTCC; for TY0708,
5′-TACTGGAAGAACTCAACGCGCTATTGTTACAAGAGGAAGCCTGACGGATGGT
GTAGGCTGGAGCTGCTTC and

5′-AAGTTTAAAGGATGTTAGCATGTTTTACCTTTATAATGATGATAACTTTTATGG GAATTAGCCATGGTCC. The amplified fragment was introduced into BW25113 harboring pKD46, which encodes λ phage Red recombinase, and chloramphenicol-resistant colonies were screened by PCR with 5′-GTCAACGATT GTTTCTGATT and 5′-ACTTTATAGT TTCCCAGTTT for TY0703, 5′-TAACGGAATA CATGGCCTGG and 5′-CGATCAAAAC CCGCATCCGT for TY0707 and 5′-TACTGGAAGA ACTCAACGCG and 5′-AGTTTAAAGG ATGTTAGCAT for TY0708 to select cells with a deleted target gene. TY0731 and TY0732 were also constructed as described above. The PCR product was introduced into O157:H7 harboring pKD46. Primers used were: for TY0731, 5′-CGTTGTAACCCGTAACTTATTTTTGCCAAAATTTTGGATACAGAATAAATGTGT AGGCTGGAGCTGCTTC and 5′-CATTTATCGTTTTATTATATATCATAATGAATTATTTTAACCTTAAATCT ATGGGAATTAGCCATGGTCC; for TY0732, 5′-TCAAACTAGGATTGAAGAGCAAATAATGATTATAATGAAGGGTAATTAAAGTG TAGGCTGGAGCTGCTTC and 5′-TGAAAAAATAGGAGTAACCGTAAATATTATTTTATCAACCATCTCGTATAATGG GAATTAGCCATGGTCC. In the deletion of *ompC* from the genome of O157:H7 wild-type, TY0731 or TY0732, the DNA fragment was amplified by PCR using JW2203 DNA as the template and the primers 5′-CCGGTACCTAAAAAAGCAAATAAAGGCA and 5′-CCAAGCTTTGTACGCTGAAAACAATG, and was introduced into O157:H7 wild-type, TY0731 or TY0732 harboring pKD46. Kanamycin-resistant colonies were screened by PCR with same primers to select TY0750, TY0751 and TY0752.

Plasmid construction and marker-rescue test

To construct pLTF, a DNA fragment containing genes *37* and *38* was amplified by PCR using T4 phage DNA as the template and the primers FR80 and FR81 (Tétart *et al.*, 1998) previously phosphorylated at their 5'-termini with T4 polynucleotide kinase and ligated into the *Eco*RV site of pBluescript II SK+. pLTF-Nik1, pLTF-Nik2, pLTF-Nik8 and pLTF-Nib, each of which has a single base substitution in gene *37*, were constructed using a KOD-Plus-Mutagenesis Kit (TOYOBO) with pLTF as the template. Primers used for mutagenesis were: for pLTF-Nik1, 5′-GAAGGTGGTAATAAGATGTCATCATATGCCATAT and 5′-ACCAGTACCATTCCATGCCTCGATG; for pLTF-Nik2, 5′-GAGGGTGGGAGTAACACTAATGCAGCAGGG and 5′-CCTGTATGATATGGCATATGATGA; for pLTF-Nik8, 5′-GTAGGTAGTAATAAGATGTCATCATATGCCATAT and 5′-ACCAGTACCATTCCATGCCTCGATG; for pLTF-Nib, 5′-TGGTATGGTGTAGGTGGTAATAAGATGTCATCA and 5′-TTCCATGCCTCGATGTAGTGGCTGT.

To construct pBRSupD, a DNA fragment containing *serU* was amplified by PCR using *E. coli* B40su1 DNA as the template and the primers 5′-AACCAGTTCAAAACGATAGG and 5′-TTTCCACTATCAACAAGGAG, digested with *Eco*RV and ligated into the corresponding site of pBR322 (Sutcliffe, 1979).

For marker-rescue test, a mutant phage, *amN91 amSE31*, which has double amber mutations in genes *37* and *38* respectively, was propagated on BW25113 cells

harboring pLTF, pLTF-Nik1, pLTF-Nik2, pLTF-Nik8, or pLTF-Nib. During propagation, each mutation in plasmid was transferred into the T4 genome via homologous recombination between plasmid and T4 DNA. The total number of progeny was calculated by plating on B40su1 or BW25113 cells harboring pBRSupD. To select recombinant phages, the progeny was plated on BB cells for pLTF, pLTF-Nik1, pLTF-Nik2, and pLTF-Nik8, and BW25113 cells for pLTF-Nib. Plaques formed on BB or BW25113 were randomly picked up and the number of plaques showing the Nik or Nib phenotype was counted by plating on BB and BW25113 cells. The emergence rate (%) shows the ratio of the number of phage plaques showing the same host range as Nik or Nib to the total number of progeny. Each value indicates the mean of three independent experiments.

References:

Cherepanov, P.P., and Wackernagel, W. (1995) Gene disruption in Escherichia coli: TcR and KmR cassettes with the option of Flp-catalyzed excision of the antibiotic-resistance determinant. *Gene* **158**: 9–14.

Datsenko, K.A., and Wanner, B.L. (2000) One-step inactivation of chromosomal genes in Escherichia coli K-12 using PCR products. *Proc Natl Acad Sci USA* **97**: 6640–6645.

Sutcliffe, J.G. (1979) Complete nucleotide sequence of the Escherichia coli plasmid pBR322. *Cold Spring Harb Symp Quant Biol* **43**: 77–90.

Tétart, F., Desplats, C., and Krisch, H.M. (1998) Genome plasticity in the distal tail fiber locus of the T-even bacteriophage: recombination between conserved motifs swaps adhesin specificity. *J Mol Biol* **282**: 543–556.

Wilson, G.G., Young, K.K.Y., Edlin, G.J., and Konigsberg, W. (1979) High-frequency generalised transduction by bacteriophage T4. *Nature* **280**: 80–82.
